# Ecological and demographic drivers of kin‐directed cooperation in a social bird: Insights from a long‐term study

**DOI:** 10.1111/1365-2656.14237

**Published:** 2025-01-28

**Authors:** Jennifer Morinay, Beth K. Woodward, Andrew F. Russell, Stuart P. Sharp, Ben J. Hatchwell

**Affiliations:** ^1^ Ecology & Evolutionary Biology, School of Biosciences University of Sheffield Sheffield UK; ^2^ Science and Technology Facilities Council UKRI Swindon UK; ^3^ Centre for Ecology and Conservation University of Exeter Cornwall UK; ^4^ Lancaster Environment Centre Lancaster University Lancaster UK

**Keywords:** helping behaviour, inclusive fitness, kin neighbourhoods, kin structure, kin‐directed cooperation, long‐tailed tit *Aegithalos caudatus*, natal dispersal, sociality

## Abstract

1. The evolution of sociality is one of the major evolutionary transitions in the history of life and a key step in this transition is the occurrence of kin associations. Yet, the question of what demographic processes and environmental factors generate kin‐structured populations and drive kin‐directed cooperation remains open.
2. In this review, we synthesise 30 years of studies of the long‐tailed tit *Aegithalos caudatus*, which has a kin‐selected cooperative breeding system with redirected help: failed breeders may help to raise offspring of conspecifics, typically relatives, breeding nearby. We describe the use of ecological, demographic, genetic and behavioural approaches to reveal: (a) how kin‐structured populations (here ‘kin neighbourhoods’) arise; (b) why the prevalence of cooperation varies among populations and individuals; and (c) how variation in dispersal and opportunities for cooperation influence individual fitness.
3. The kin neighbourhoods of long‐tailed tits arise from three processes. First, natal dispersal is limited and sex‐biased so many individuals, especially males, recruit as breeders close to their natal site. Second, neither dispersal nor migration necessarily disrupts kin associations because long‐tailed tits often move with close relatives. Third, a small effective population size driven by high nest predation rates enhances within‐population relatedness. Together, these processes set the scene for kin‐directed helping behaviour by causing spatial clustering of relatives.
4. The prevalence of cooperation within kin neighbourhoods depends on several factors, both at the population‐level (annual nest predation rate and length of the breeding season) and individual‐level (relatedness, familiarity, sex and condition). However, limited information on prior social association and the reliability of kin discrimination cues hampers our current understanding of individual helping decisions.
5. Finally, variation in dispersal within and between sexes affects the probability of interacting with kin, the likelihood of cooperation, and accrual of the direct and indirect components of inclusive fitness.
6. We use this comprehensive understanding of the factors driving cooperative behaviour in long‐tailed tits to highlight gaps in knowledge and suggest future avenues for research in this system, and to make general inferences about the role of dispersal, demography and kinship in social evolution.

## Graphical abstract

We review 30 years of studies on the drivers of kin structure and cooperative behaviour in an atypical cooperative breeder, the long‐tailed tits. These numerous studies have nurtured and developed keystone ideas on the evolution of sociality and cooperation. Picture credit: Billy Clapham.

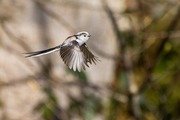

## 1 INTRODUCTION

The evolution of sociality is one of the major evolutionary transitions in the history of life on earth, opening up numerous ecological and behavioural opportunities that are unavailable to non‐social organisms (Bourke, 2011). The development of social evolution theory has been greatly influenced by the concept of relatedness arising from population viscosity (Hamilton, 1964), and empirical studies show that sociality has evolved most frequently in kin‐structured populations where social interactions among relatives are frequent and kin selection can operate (Rubenstein & Abbot, 2017). Therefore, understanding how kin structure arises in populations is fundamental to explaining the evolutionary transition to sociality and cooperation.

Dispersal is important in many aspects of ecology because of its critical role in processes such as population dynamics, local adaptation and inbreeding avoidance (Clobert et al., 2012). In a social context, dispersal determines gene flow and hence the genetic structure of populations (Hamilton, 1964), so the factors constraining dispersal and driving family formation have been prominent in the development of ideas on the evolution of cooperative breeding in vertebrates (Clutton‐Brock & Lukas, 2012; Griesser et al., 2017). In birds, this focus has led to the proposal of various factors that might enhance population viscosity, such as the ecological constraints hypothesis (Emlen, 1982), the benefits of philopatry hypothesis (Stacey & Ligon, 1991) and the life history hypothesis (Arnold & Owens, 1998). Large‐scale comparative analyses (e.g. Jetz & Rubenstein, 2011) suggest that unpredictable or harsh environments may be a common factor driving social transitions, but such effects are minor relative to phylogenetic effects and lack predictive power (Cockburn et al., 2017). Moreover, while the formation of family groups appears to be a key step in the transition to cooperative breeding in birds, it is not a definitive one because not all family living species breed cooperatively (Drobniak et al., 2015).

One issue highlighted by such comparative studies is that cooperatively breeding species exhibit diverse social systems including cooperative polygamy (e.g. dunnocks *Prunella modularis*, Davies, 1992), systems with joint‐nesting pairs (e.g. Taiwan yuhinas *Yuhina brunneiceps*, Shen et al., 2016) and co‐breeding (e.g. acorn woodpeckers *Melanerpes formicivorus*, Koenig et al., 2016), as well as the typical non‐breeding helper‐at‐the‐nest system (e.g. Florida scrub‐jays *Aphelocoma coerulescens*, Fitzpatrick & Bowman, 2016). Thus, rather than being a homogeneous phenomenon with groups forming through delayed dispersal of offspring, there is considerable variation in the mode of group formation, reproductive skew, the sex and relatedness of group members, and the age structure of social groups (Ben Mocha et al., 2023; Cockburn et al., 2017). Unsurprisingly, such diverse social systems may arise via multiple routes that involve variable dispersal strategies.

In most kin‐based cooperative breeding systems, offspring delay dispersal and, while they remain in the family group, help the breeders raise subsequent broods (Hatchwell, 2009). In these systems, helpers have little choice of whom to help. In an intriguing fraction of cooperatively breeding birds, however, breeders may redirect their help after breeding failure (e.g. western bluebirds *Sialia mexicana*, Dickinson et al., 2016; rifleman *Acanthisitta chloris*, Preston et al., 2013). When failed breeders become helpers, they often have a choice of several potential recipients, related and unrelated, to whom they can redirect their care. Importantly, cooperative systems with redirected help share one important trait with other cooperative species, which is that cooperation most often occurs among relatives (Dickinson & Hatchwell, 2004). However, they do not differ fundamentally from non‐cooperative species either, because juveniles undertake natal dispersal to become breeders before any helping opportunities. Therefore, such systems may represent an intermediate stage in the transition to sociality (Ligon & Burt, 2004). They also provide an ideal situation for investigation of kin preferences in helping, and the fitness consequences of choosing to help kin or non‐kin.

**FIGURE 1:**
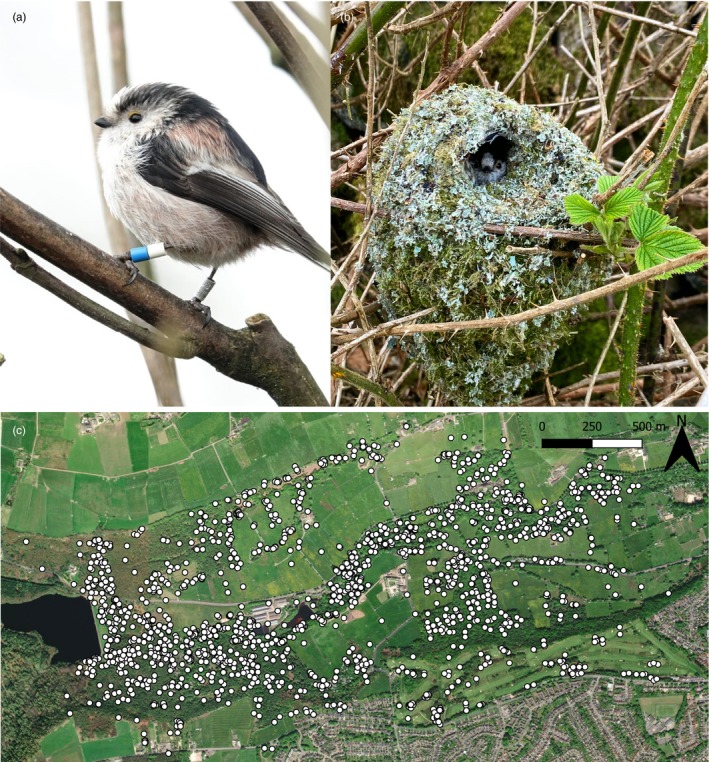
Ringed adult long‐tailed tit (a) perched and (b) in its nest. (c) Monitored nests in the Rivelin Valley in 1994–2023. Credits: (a) Bob Russon, (b) Sarah Biddiscombe.

**FIGURE 2:**
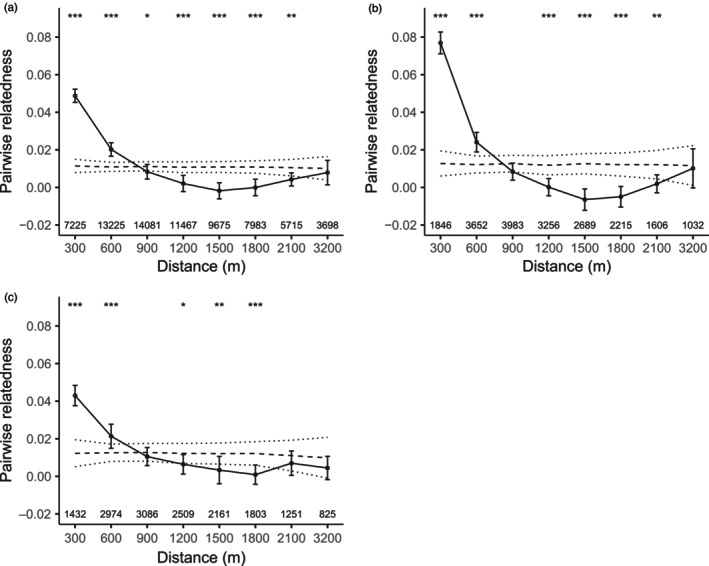
Mean pairwise genetic relatedness of breeders in Rivelin Valley (UK) over increasing distances between compared nests. Solid lines: Estimates of pairwise relatedness among (a) all individuals, (b) males, and (c) females. Error bars: SE estimates from jackknifing over loci. To determine whether these observed relatedness estimates were significantly different from random, Leedale et al. (2018) compared them to the null estimates represented by dashed lines: Average relatedness and 95% CI in a simulated unstructured population obtained from random permutations. Statistically significant differences between the observed and null estimates: *p < 0.05, **p < 0.01, ***p < 0.001. From Leedale et al. (2018).

Here, we review the findings of a long‐term study of long‐tailed tits *Aegithalos caudatus* (Figure 1) that has investigated the factors driving the emergence of kin‐structured populations and helping behaviour. Long‐tailed tits live in kin neighbourhoods (Figure 2) and have a cooperative breeding system in which failed breeders redirect their care to help raise the broods of neighbours (Glen & Perrins, 1988). Help is a kin‐selected behaviour, with helpers accruing indirect fitness benefits but no direct fitness benefits from their cooperation (Hatchwell et al., 2014). First, we review studies of the demographic and life history traits generating kin neighbourhoods, focusing especially on the role of natal dispersal. Second, we review studies investigating the factors that determine the prevalence of helping behaviour in kin neighbourhoods, considering both individual and population‐level factors. Finally, we focus on studies examining the fitness consequences of variation in natal dispersal distances within and between sexes, showing that contrasting sex‐specific patterns of fitness accrual drive divergent selection on this key demographic trait. We conclude by identifying gaps in our knowledge of what drives helping decisions in long‐tailed tits and by addressing the broader implications of these findings for our understanding of social evolution.

## 2 A MODEL SYSTEM IN SOCIAL EVOLUTION

### 2.1 Behavioural ecology of the long‐tailed tit

The long‐tailed tit is a small (7–8 g) insectivorous and sexually monomorphic passerine distributed from western Europe to Japan. In the United Kingdom, long‐tailed tits are sedentary with fission‐fusion social dynamics. Outside the breeding season, they live in flocks of c. 10–20 birds with large overlapping ranges (Hatchwell et al., 2002). Flocks usually comprise juveniles and adults of both sexes that may be philopatric or immigrants, and their composition is fluid, with birds switching between flocks and occasional mergers occurring. Consequently, flocks are normally composed of related (two or more nuclear families) and unrelated birds (Napper & Hatchwell, 2016). Flocks break up in late winter with all individuals forming pairs and starting nest‐building in early March. Nestling and adult sex ratios are typically even (Nam et al., 2011) and all birds attempt to breed from their first year onwards. Both sexes build nests, but females incubate the clutch alone (typically 8–11 eggs). The brood hatches synchronously and both parents feed the offspring until c. 3 weeks after fledging. Long‐tailed tits are single‐brooded, with few nests initiated after early May at our study site. See Hatchwell (2016) for further details.

Long‐tailed tits are cooperative breeders with a redirected helping system in which failed breeders may care for the brood of other pairs. Most nests fail due to depredation of eggs or chicks by corvids or mustelids. If failure occurs early in the season, pairs re‐nest; but should failure occur after early May, breeders abandon breeding for that year, and some become helpers (MacColl & Hatchwell, 2002). Provisioning of nestlings and fledglings is the main contribution of helpers in this system, although they may occasionally provision incubating females earlier in the breeding cycle (3.9% of 179 helpers in our study population, Hatchwell et al., 2004). Around one quarter of failed breeders become helpers, males being more likely to redirect their care than females (38% and 9% of male and female failed breeders becoming helpers respectively; Sharp et al., 2011). Therefore, most helpers are male (81%, *n* = 177 helpers; Leedale et al., 2018), and they are typically related to the male breeder (most often a brother, Nam et al., 2010). Overall, helpers mostly redirect their care to kin: 57% of helped pairs included a breeder that was a first‐order relative of the helper, 13% of helped pairs contained a second‐order relative, and 30% of pairs were unrelated (*n* = 181 helpers, based on genetic relatedness; Leedale et al., 2018). The mean relatedness of helpers to the brood they cared for was 0.20 when assisting kin, 0 when assisting non‐kin, and 0.17 on average (Hatchwell et al., 2014). Kin‐directed help does not simply result from population viscosity, that is from the fact that nests of kin are available in the vicinity but also from active kin preference. First‐order kin (parent, offspring or siblings, categorised either genetically or from the social pedigree) were helped significantly more than random expectation (Leedale et al., 2018), and individuals that were experimentally provided with the choice to help at equidistant nests of kin and non‐kin favoured kin (Russell & Hatchwell, 2001).

Fledglings from helped broods had a higher recruitment probability (Hatchwell et al., 2004), each helper at a nest increasing the probability that a male fledgling recruited locally by 6%–8% (recruitment probability, in %: 0 helpers: 16%; 1 helper: 22%; 2 helpers: 28%; 3 helpers: 35%; 4 helpers: 43%; Hatchwell et al., 2014). Helpers therefore accrue indirect fitness providing they help relatives (Hatchwell et al., 2014). In contrast, previous analyses detected no direct fitness benefit of helping, in terms of survival, or current or future breeding prospects (Meade & Hatchwell, 2010). Helpers had a higher survival rate than non‐helpers, but this resulted from helping decisions being condition‐dependent: only individuals in good condition, with helping opportunities available nearby, became helpers (Meade & Hatchwell, 2010). Indeed, helping was costly for future survival when controlling for this effect (Hatchwell et al., 2014). Regarding helpers' reproductive opportunities, Meade and Hatchwell (2010) found no effect of helping on future productivity, and contrary to many other cooperative systems (Downing et al., 2018), there was no possibility of direct reproductive benefits in the current season via inheritance of territory or mate in this single‐brooded and non‐territorial species. In addition, helpers very rarely associated with pairs before the nestling period (Hatchwell et al., 2004), so they had limited fertilisation or egg‐laying opportunities. Green and Hatchwell (2018) found that intraspecific brood parasitism was negligible, and extra‐pair paternity was low: 10% ± 8 SD of 1957 nestlings (yearly range: 0%–29%), originating from 27% ± 18 SD of 254 broods (yearly range: 0%–63%), were extra‐pair offspring (1995–2015 data). In a smaller sample of broods, mate‐switching was not responsible for these mixed paternities, which were instead attributed to extra‐pair copulations (Hatchwell et al., 2002). Assigning paternity to extra‐pair offspring when potential fathers are related is challenging (Marshall et al., 1998), but three lines of evidence indicate that helpers achieved very little direct fitness via extra‐pair paternity and that unrelated helpers did not target their care towards broods containing their extra‐pair offspring. First, when helpers (*n* = 15) fed a brood belonging to unrelated breeders, the mean relatedness coefficient of the helper to the brood was not significantly different from 0 (−0.07 ± 0.02 SE, *n* = 15; Nam et al., 2010). Second, Green and Hatchwell (2018) reported that just 2.9% (5/173) of recruits into the study population with assigned paternity were the offspring of helpers. Third, in a sample of 28 helpers, just 6 (21%) were assigned as parents of any offspring in the helped brood (Hatchwell et al., 2002). A number of such cases were expected by chance because extra‐pair males were usually close neighbours (Hatchwell et al., 2002), and helpers were also close neighbours of the nest where they eventually helped (Leedale et al., 2018). Additionally, the low level of extra‐pair paternity and high frequency of cooperation (c. 50% of successful nests have helpers) suggest that gaining direct fitness benefits by helping to provision broods containing extra‐pair offspring is an unlikely mechanism for the evolution of cooperation in this system (Hatchwell et al., 2002). Note that extra‐pair paternity was accounted for when estimating individual fitness (e.g. Green & Hatchwell, 2018). In addition, we used social pedigrees as well as genetic relatedness when investigating kinship effects on helping because evidence indicates that the birds themselves have access only to social information about relatedness (e.g. Leedale et al., 2018; Nam et al., 2010).

This cooperative breeding system is, therefore, likely to be the product of kin selection, with helping behaviour satisfying Hamilton's rule (Hatchwell et al., 2014). Moreover, the timing of the switch from a breeding to helping strategy is consistent with a seasonal decline in reproductive success, the expected indirect fitness pay‐off from helping exceeding that of breeding late in the season (MacColl & Hatchwell, 2002).

Long‐tailed tits are ideal for investigating the factors affecting dispersal decisions and their consequences for kin‐structure and cooperation. First, their kin‐selected redirected helping behaviour is only possible because relatives are locally available. Therefore, while natal dispersal occurs in birds' first winter, there must be demographic features generating kin neighbourhoods. Second, the estimated survival of long‐tailed tit fledglings was 42% (Sharp, Baker, et al., 2008; Sharp, Simeoni, & Hatchwell, 2008) with local recruitment of 20% (see Section 3.1), facilitating investigation of dispersal decisions even in open populations. Third, this species is short‐lived with approximately 50% adult mortality per annum (McGowan et al., 2003), meaning that life histories for a large sample of birds could be acquired relatively quickly, allowing estimation of lifetime reproductive success. Fourth, long‐tailed tits have no age‐related changes in behavioural strategy or survival, with individuals readily switching back and forth between breeding and helping (Roper et al., 2022), unlike the complex age‐structured life histories of many cooperatively breeding species (Cockburn et al., 2017). Finally, their simple cooperative breeding system, with no direct fitness benefits of helping detected, makes dissection of the direct and indirect fitness consequences of dispersal decisions relatively straightforward.

### 2.2 Study population, phenology and nest monitoring

We studied a population of c. 17–82 pairs of long‐tailed tits occupying a c. 3 km^2^ study site in the Rivelin Valley Sheffield, UK (53°23′ N, 1°34′ W) from 1994 to 2023 (Figure 1). The Rivelin Valley is a patchwork of primarily oak and birch woodland, with areas of open farmland and scrub, and a golf course. The area is bounded by open moorland and fields for c. 50% of its perimeter, but the habitat is continuous with suburban gardens and woodland for the remainder. Thus, the population is open with c. 40% of breeders in each year being immigrants from outside the study area.

Every year we followed the same protocol for intensive monitoring of life histories and behaviour, except in 2001 when access to the study site was restricted. In brief, their intricate dome‐shaped nests, which are built of moss bound together with spider silk, covered with thousands of lichen flakes and lined with feathers, were found by following pairs during nest‐building. It was estimated that c. 95% of all nests built in the study area were located, those missed being short‐lived attempts that were abandoned or depredated early in the breeding cycle. Nests were usually in vegetation ≤3 m from the ground (71%, 734/1031) and so could be closely monitored. For these nests, we recorded first egg lay date, clutch size, hatch date and fledge date. Inaccessible nests (high in trees) were monitored by observing breeders' behaviour, allowing estimation of first egg lay date, clutch completion date, hatch and fledge dates. If a breeding attempt failed, we searched for replacement nests nearby. To identify helpers and estimate carers' provisioning rates, we observed all nests with nestlings for 1 h every second day, weather permitting, from day 2 (hatching = day 0) until fledging (day 16–18) or nest failure.

To compare results obtained in the Rivelin Valley population with other populations differing in key ecological features (specifically, dispersal and migratory behaviour), we also report observations following very similar protocols, conducted at Melton Wood, Doncaster, UK (53°20′ N, 1°30′ W) in 2001–03 and in Nigula National Park, Estonia (59°00′ N, 24°40′ E) in 2005–07.

### 2.3 Captures, genotypes and dispersal

Every year c. 95% of all adults in the population were uniquely marked with two colour‐rings and a British Trust for Ornithology (BTO) ring (under BTO licence). Adults were captured using mist‐nets, with standard biometrics (body mass, wing and tarsus length) recorded at each capture. Nestlings in accessible nests were ringed on day 11 with unique combinations of two colour‐rings and a BTO ring, and body mass and tarsus length were recorded. Permission to ring adult and nestling long‐tailed tits with a British Trust for Ornithology (BTO) ring and two colour‐rings was granted under licence from the BTO to BJH (BTO licence C3770; project licence 4719). Blood samples were taken upon each individual's first capture by brachial venepuncture under licence from the UK Home Office to BJH (project licence PPL—PP5912664; personal licence PIL IE73AE8C8). All procedures used in the project were approved by the Animal Welfare and Ethical Review Body of the University of Sheffield. Individuals were sexed and genotyped at up to 19 microsatellite loci following standard protocols (Simeoni et al., 2007). Coefficients of relatedness calculated from microsatellite genotyping provided a close approximation of pedigree relatedness (Nam et al., 2010). Parent‐offspring and full‐sibling relationships could not be distinguished from microsatellite genotyping alone, but they could be using social pedigree of individuals with known life‐histories. Unringed breeders or helpers that appear in the population were assumed to be 1‐year old immigrants unless they could be genetically assigned as the offspring of a pair that fledged an unringed brood of chicks from a successful but inaccessible nest in the previous year. The rationale for this assumption is that almost all adult breeders/helpers were marked, and dispersal of breeders was very limited, with an annual resighting probability of 83%–100% for marked adults (Gullett et al., 2014; McGowan et al., 2003).

## 3 ECOLOGICAL AND DEMOGRAPHIC PROCESSES PROMOTING KIN NEIGHBOURHOODS

Kin structure sets the stage for cooperative behaviour to emerge in long‐tailed tits, so determining the processes leading to it is the first step towards understanding the evolution of sociality. Over the course of this long‐term study, we identified three key ecological and demographic mechanisms in long‐tailed tits that together generate kin neighbourhoods: limited natal dispersal, coordinated dispersal of relatives and a small effective population size.

### 3.1 Limited natal dispersal

**FIGURE 3:**
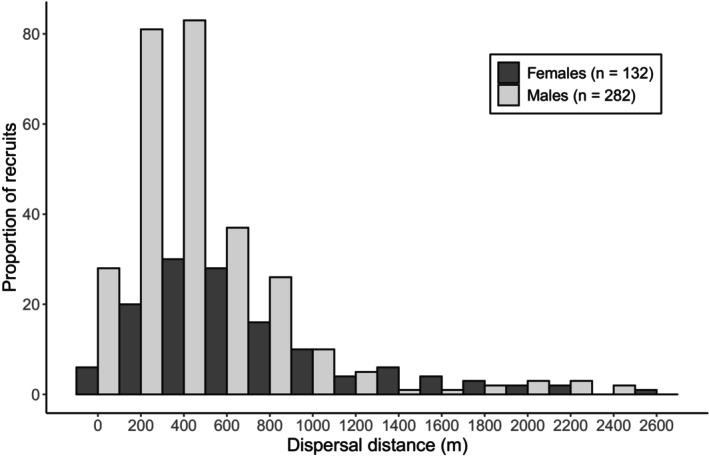
Distribution of natal dispersal distances for philopatric female (darker) and male (lighter) long‐tailed tits in Rivelin Valley born in 1995–2022. These data were not previously published.

Natal dispersal is the net movement of an individual between its natal site and first breeding location. Dispersal typically dilutes kinship, so delayed natal dispersal is considered a prime factor generating kin structure in cooperative species (Emlen, 1982; Koenig et al., 1992). Long‐tailed tits are atypical cooperative breeders because virtually all first‐year birds in our study population disperse and become breeders. Yet, many disperse only short distances. For cohorts born in 1995–2022, local recruits dispersed on average 534 m ± 458 SD (*n* = 438) and although both sexes often recruited in the study site, dispersal was female‐biased (Figure 3). Accordingly, the recruitment of ringed fledglings within the study area between 1995 and 2021 was 20.0% (*n* = 4072 fledglings from 325 broods), and was higher for males (24.8%, *n* = 2107 from 317 broods) than females (14.6%, *n* = 1907 from 315 broods; note that 58 fledglings were not sexed; original data). Note that many females and some males must disperse beyond the boundaries of our study site, so these are minimal estimates (Sharp, Baker, et al., 2008; Sharp, Simeoni, & Hatchwell, 2008). The short dispersal distances and high recruitment rate of long‐tailed tits increased the probability of breeding close to kin. There was stronger kin structure among males than females, but post‐dispersal fine‐scale genetic structure existed in both sexes, relatedness among adults living within 600 m being significantly higher than expected (Figure 2; Leedale et al., 2018). Long‐tailed tits sought helping opportunities within 400 m of their failed nest (mean = 335 m ± 262 SD for 164 males, 406 m ± 347 for 37 females), so many adults would have at least one first‐order relative (relatedness = 0.5) breeding within this distance (Leedale et al., 2018).

These studies suggest that kin neighbourhoods could simply result from long‐tailed tits' limited dispersal. However, limited dispersal is not unusual among temperate passerines (Paradis et al., 1998) and many non‐cooperative species living in comparable habitats disperse similar distances (Russell, 1999). These interspecific comparisons raise the possibility that other demographic and/or life history mechanisms contribute to genetic structure in long‐tailed tit populations.

### 3.2 Coordinated natal dispersal

Dispersal is usually an obstacle to the emergence of kin structure through its disruptive effect on social bonds among relatives. Yet, dispersal may not disrupt kin associations if relatives disperse together, as observed in several cooperative breeders (e.g. Dawson Pell et al., 2021; Koenig et al., 2000; Wang & Lu, 2014). Studying relatedness among dispersers is challenging in open populations because dispersers disappear from the study area and immigrants are usually unmarked individuals of unknown history. However, using microsatellite genotype data from breeding long‐tailed tits in 1994–2005, Sharp, Simeoni, and Hatchwell (2008) estimated relatedness among genetically confirmed immigrants.

Immigrant long‐tailed tits formed coalitions of related dispersers; over half of all immigrants belonged to flocks containing two to seven full siblings (Sharp, Simeoni, & Hatchwell, 2008; full sibling and parent‐offspring relatedness could not be discriminated genetically, but immigrants were assumed to be in their first year, i.e. siblings). These coalitions were sex‐biased, with significant deviation from a 1:1 sex ratio across the 37 groups of 2–7 relatives (mean = 2.9 ± 1.13 SD), including 14 all‐female and 4 all‐male groups. Strong kin associations among immigrants created opportunities for kin‐directed helping because whether an immigrant had dispersed with relatives or not influenced its propensity to help after breeding failure: those that immigrated with at least one sibling tended to become helpers (32%, *n* = 22) more often than those that immigrated without siblings (12%, *n* = 34; Sharp, Simeoni, & Hatchwell, 2008).

These coordinated dispersal events also occurred for philopatric individuals dispersing within the study area, further structuring the population at a fine scale. Philopatric (especially same‐sex) siblings tended to disperse similar distances, in the same direction (1994–2004; Sharp, Baker, et al., 2008). Such same‐sex sibling associations during dispersal, both at fine and broad scales, may allow long‐tailed tits to reduce inbreeding risks while maintaining kin neighbourhoods (Leedale, Simeoni, et al., 2020).

The ability of long‐tailed tits to retain kin associations through dispersal suggests that migration may not be incompatible with kin‐selected cooperative breeding providing that family ties persist during migration. The latter has been reported in several large‐bodied species such as white‐fronted geese *Anser albifrons* (Weegman et al., 2016) and tundra swan *Cygnus columbianus* (Scott, 1980) and could exist in small passerines too. This possibility led us to investigate whether kin‐directed helping occurred in a migratory population of long‐tailed tits and, if so, how kin association during breeding is achieved despite long distance migration.

Woodward (2008) studied a migratory population of long‐tailed tits in Nigula National Park, Estonia, showing that they have a kin‐directed helping system similar to that observed in the UK (Table 1). But how do migratory long‐tailed tits retain kinship ties enabling them to favour kin when helping? Chetverikova et al. (2017) have subsequently found that 61%–73% of long‐tailed tits on southward migration in the autumn in northern Europe and eastern Asia travelled with at least one relative, but in her unpublished PhD thesis Woodward (2008) aimed to test whether kin associations were maintained throughout the migratory period, as observed at a local scale in the Rivelin Valley.

**TABLE 1:** Characteristics of study populations and helping behaviour in the Rivelin Valley (original data) and the Nigula National Park (data from Woodward, 2008) populations of long‐tailed tits.

	Rivelin Valley (UK)	Nigula National Park (Estonia)
Study site		
Size (km^2^)	3	26.5
Study years	1994–2023	2005–2007
Breeding density (pairs/km^2^)	17.9	0.8
Predation rate (*n* nests)	63% (1570)	72% (61)
Helping behaviour		
Number of detected helpers	415	26
Successful nests (*n*) with helpers	49% (444)	80% (10)
Number of helpers per helped nests	1.77 ± 1.05 SD	1.53 ± 0.77 SD
Helpers that are males	81%	75%
Helping ≥1 second order kin (*r* ≥ 0.25)	71%	81%
Helping ≥1 first order kin (*r* = 0.5)	59%	31%

On southward migration, 27 entire flocks (mean = 12.6 birds/flock) were caught at Kabli Ringing Station, Estonia (58°01′ N, 24°45′ E) in September 2005. Sixteen of these flocks were recaptured with the same flock‐mates shortly afterwards: eight at Ventes Ragas, Lithuania (331 km south‐west of Kabli) and eight at Fringilla, Russia (398 km south‐west of Kabli). On northward migration, seven flocks (mean = 7.4 birds) were captured at Rybachy Biological Station, Kaliningrad, Russia (55°09′ N, 20°51′ E) in March 2007. Microsatellite genotyping revealed that 82% of flock members (*n* = 27 flocks) migrated with at least one first‐order relative (relatedness = 0.5, parent‐offspring or full‐sibling relationship) in autumn and 57% (*n* = 7 flocks) returned with one or more first‐order relatives in spring. These estimates of kin composition of migratory flocks of long‐tailed tits in the eastern Baltic were even higher than equivalent estimates in sedentary flocks in Rivelin Valley. There, based on a combination of social pedigrees and sibship reconstruction, 44% of birds in autumn flocks (October 1; *n* = 35 flocks, mean = 7.9 birds/flock) and 39% of birds in spring flocks (February 1; *n* = 35 flocks, mean = 6.7 birds/flock) had at least one first‐order relative in the same flock (Woodward, 2008). Thus, remarkably, long‐tailed tits can retain kinship ties throughout the winter despite undertaking long‐distance migratory movements, thereby facilitating kin‐directed helping behaviour similarly to that observed in sedentary populations.

Cooperative breeding is extremely rare in migratory species (Arnold & Owens, 1998), and the few documented cases tend to be in colonial species, such as the European bee‐eater *Merops apiaster* (Lessells, 1990), rainbow bee‐eater *Merops ornatus* (Boland, 2004) and dusky wood‐swallow *Artamus cyanopterus* (Sims, 2007), where the spatial distribution of nests within a colony may provide cues to kinship. Arguably, strong site fidelity could achieve the same result in long‐tailed tits. However, spatial cues alone are insufficient to allow reliable kin‐directed help in sedentary populations (Leedale et al., 2018), let alone in migratory ones, but coordinated movements and continuous kin‐association outside the breeding season in both sedentary and migratory populations clearly facilitate such behaviour. To address this problem, we are currently investigating whether related birds in the Rivelin Valley must spend the winter together (i.e. in the same flock) to recognise each other in a following year, or whether they can be separated and still remember their relatives.

### 3.3 Small effective population size

Population viscosity—limited or delayed dispersal—is regarded as the main mechanism generating kin‐structured populations of social species, but little empirical attention has been devoted to other demographic features that may have similar effects. Lehmann and Balloux (2007) proposed that fecundity bias (high variance in fecundity) may lead to relatively few ancestors for the next generation (i.e. a small effective population size) and hence increased relatedness among recruits, favouring the emergence of kin‐selected cooperation (Lehmann & Rousset, 2010).

One ecological driver of such fecundity bias is predation, which may impact groups rather than individuals. In birds, most species produce multiple offspring that are spatially confined in early life so predators can target entire broods. Yet, studies demonstrating the importance of the timing and target of predation for structuring the populations of social species are scarce. In long‐tailed tits, full brood predation is the commonest cause of reproductive failure (Hatchwell et al., 2013), and since helpers enhance productivity, fledgling recruitment rate is notably higher in some nests than others (Sharp, Baker, et al., 2008). Thus, some breeders must contribute many more offspring to the next generation than others.

To investigate the effect of fecundity bias on the kin structure of long‐tailed tits, Beckerman et al. (2011) developed an individual‐based model, parameterised to mimic alternative mortality regimes: offspring mortality targeting family groups (clustered, pre‐fledging predation) or individuals (post‐fledging predation). The proportion of close kin recruiting and the mean and variance in relatedness should be higher when predation targets family groups rather than individuals. To disentangle this effect of fecundity bias from possible effects of dispersal and adult survival on relatedness, both mortality regimes were modelled while accounting for immigration, emigration, local recruitment, and adult survival. Thirteen years of data informed parameter settings in the models, which simulated 40 years of demographic and dispersal processes.

As predicted, the offspring mortality regime affected relatedness over and above the effects of population viscosity: mean relatedness, variance in relatedness and number of kin (relatedness ≥0.25) were higher in simulations when mortality was clustered within families than when it affected individuals. The effect of fecundity bias on kin structure was independent of any benefits/costs of interacting with kin. However, the emergence of kin‐directed behaviour, facilitated by this kin structure, would be expected to initiate a positive feed‐back loop which would further distinguish the effect on relatedness of the two mortality regimes. On the other hand, predation of broods is a common reason for nest failure in birds, many species exhibiting strongly skewed reproductive success (Clutton‐Brock, 1988; Newton, 1989), so the relative importance of predation regime as a driver of kin structure and the evolution of cooperation remains unclear.

## 4 FACTORS PROMOTING HELPING BEHAVIOUR

### 4.1 Population‐level factors

In most avian cooperative breeders, helping is facultative and factors other than kin structure are required to drive the emergence of cooperative behaviour. In this section, by reviewing and synthesising the conclusions from previous studies conducted across years and populations, we examine what population‐level ecological and demographic factors influence the level of cooperation in long‐tailed tits.

In species with redirected helping, the prevalence of helping should depend, in part, on the probability that breeders join the pool of potential helpers by failing to breed themselves, and the availability of nests belonging to a relative where they can help. Following this reasoning, Hatchwell et al. (2013) investigated the proximate factors influencing the level of cooperation in the population, measured as helper prevalence (proportion of helpers in the population), and helping intensity (number of helpers at helped nests) across years from 1995 to 2011. Four population–level parameters were identified as potential drivers of variation in helping: (i) nest predation rate, which determines the number of failed breeders joining the pool of potential helpers, and of non‐predated nests available to receive help; (ii) length of the breeding season, which determines the time available for independent breeding and hence the probability of raising one's own brood; (iii) population size, because at high population density, competition may reduce the chance of successful breeding, leading more individuals to become helpers; (iv) an index of relatedness in the population (proportion of marked adults with at least one first‐order relative in the breeding population, assessed from the social pedigree), because opportunities for kin‐directed helping should increase with relatedness.

**FIGURE 4:**
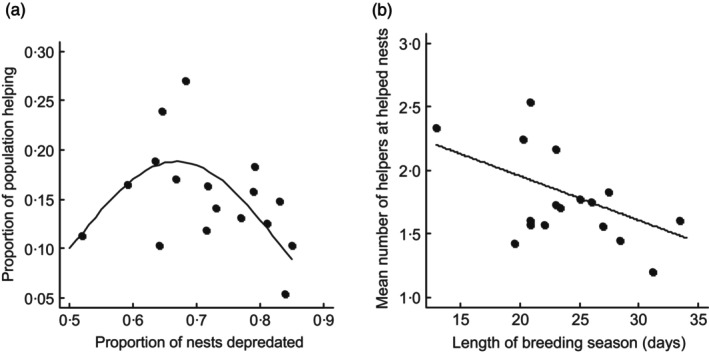
Relationship between helping metrics and (a) predation rate, and (b) length of the breeding season in the Rivelin Valley population in 1995–2011. Dots correspond to raw data and lines to models' predictions. Other variables in the model were set to their medians. Model selection was based on AICc and a model averaging approach. For the models fitting the proportion of the population helping, models with a quadratic predation effect showed stronger support than those with a simple linear effect. From Hatchwell et al. (2013).

First, helper prevalence peaked at intermediate levels of nest predation (Figure 4a), when predator pressure produced potential helpers, but was not so strong that there were few active nests available to help. Second, helping intensity was stronger during short breeding seasons, when fewer pairs renest after failure and more birds become potential helpers (Figure 4b). Third, population size did not affect helping metrics, which was unsurprising because long‐tailed tits are non‐territorial and live at quite low density so breeding opportunities should not be density‐dependent. Finally, contrary to expectations, mean annual relatedness had no significant effect on helping metrics (Hatchwell et al., 2013).

Similar conclusions were reached in another analysis of helping and relatedness in two populations that differed demographically and hence in genetic structure (Sharp et al., 2011). The two study sites, located 27 km apart, comprised predominantly deciduous woodland and scrub, but Melton Wood (MW) was surrounded by extensive farmland, while the Rivelin Valley (RV) site was partially contiguous with habitat suitable for long‐tailed tits. Sharp et al. (2011) investigated whether the difference in landscape connectivity drove inter‐population differences in dispersal, population genetic structure and cooperative behaviour.

As expected, juvenile recruitment rate was higher in MW (26.5%, *n* = 236) than in RV (14.3%, *n* = 115), a difference driven primarily by females recruiting locally in MW. Consequently, adults in MW had more close relatives than in RV (Table 2). Mean annual breeding success was similar across populations (nests producing fledglings: RV 33.6%; MW 35.5%), so the number of potential helpers and recipients were comparable. The critical question was whether higher population relatedness in MW than in RV led to more helping. Surprisingly, there was no statistically significant difference in the proportion of failed breeders that helped, the proportion of successful nests with helpers, the mean number of helpers per helped nest, the sex ratio of helpers, nor the relatedness of helpers to breeders across populations (Table 2; Sharp et al., 2011).

**TABLE 2:** Mean annual values of genetic relatedness and helping metrics in Rivelin Valley (1996–2005) and Melton Wood (2001–2003) populations of long‐tailed tits.

	Rivelin Valley	Melton Wood
Proportion of adult dyads with *r* ≥ 0.25		
All dyads	7.0% (218.2/3350.6)*	10.6% (623.0/6071.3)
Male dyads	7.8% (70.9/964.1)*	11.8% (178.0/1554.3)
Female dyads	6.6% (44.5/706.2)*	9.8% (137.3/1457.0)
Mean ± SE helper‐breeder relatedness		
Male breeder	0.190 ± 0.024	0.150 ± 0.049
Female breeder	0.057 ± 0.021	−0.02 ± 0.038
Proportion of failed breeders that became helpers		
Overall	22.9% (14.3/62.5)	17.5% (16.3/93.3)
Males	37.9% (10.8/28.5)	26.6% (12.3/46.3)
Females	8.5% (2.1/24.7)	9.5% (3.7/39.0)
Proportion of nests that had helpers	43.2% (9.5/22.0)	37.0% (11.0/29.7)
Annual sex‐ratio of helpers (*n*_males_/*n*_females_)	83.7% (10.8/12.9)	76.9% (12.3/16.0)
Annual number of helpers per helped nest (mean ± SD)	1.71 ± 0.40	1.66 ± 0.39

*Note*: We provide the mean sample sizes across years in parentheses. Stars indicate statistically significant difference. Data from Sharp et al. (2011).

These population‐level comparisons across years and sites show that, contrary to expectations, population‐level relatedness did not predict helping behaviour. Instead, decisions to help or not may be better considered at the individual level, and here we have identified additional drivers of helping.

### 4.2 Individual‐level factors

First, as described above, helpers preferentially directed their care towards relatives (Leedale et al., 2018; Russell & Hatchwell, 2001). Moreover, they provisioned broods to which they were more closely related at higher rates than those to which they were more distantly or unrelated (Leedale, Lachlan, et al., 2020; Nam et al., 2010). However, those studies also showed that c. 30% of helpers directed their care at nests of unrelated breeders and nestlings (male helpers 24%, *n* = 144; female helpers 46%, *n* = 33; Leedale et al., 2018), even though no direct benefits of helping have been detected. Hatchwell et al. (2014) argued that such costly helping decisions originate from recognition errors, a suggestion supported by Leedale, Lachlan, et al.'s (2020) analysis of potential recognition cues. Long‐tailed tits can discriminate kin from non‐kin using acoustic cues, experimental evidence showing that they behaved differently to playback of the calls of kin or non‐kin (Hatchwell et al., 2001; Sharp et al., 2005). Individual and family specific cues are embedded in the frequency parameters of calls used in social and agonistic contexts that develop at the nestling stage (Sharp & Hatchwell, 2005, 2006), and that are learned from carers (parents and helpers) rather than being innate (Sharp et al., 2005). However, call similarity as a cue to kinship is probably neither error‐proof, nor the only kin recognition mechanism used by long‐tailed tits. Leedale, Lachlan, et al. (2020) showed that helpers assisted individuals with more similar calls, but also that there was overlap in the bioacoustic features of kin and non‐kin calls. When helpers assisted unrelated breeders, the calls of those breeders were as similar to the helper's as those of their relatives, suggesting mistaken identity (Leedale, Lachlan, et al., 2020). Helpers also adjusted their provisioning effort according to their relatedness to breeders (Leedale, Lachlan, et al., 2020; Leedale, Li, & Hatchwell, 2020; Leedale, Simeoni, et al., 2020; Nam et al., 2010), but not to their call similarity with them, suggesting that other phenotypic cues may play a role in kin discrimination. For example, olfaction is used for kin recognition in other avian systems (Bonadonna & Sanz‐Aguilar, 2012; Caspers et al., 2017), a possibility that requires further research in social species, such as the long‐tailed tit.

Second, nest proximity influenced helping decisions because failed breeders helped at nests close to their last breeding attempt (Leedale et al., 2018; Sturrock et al., 2022). Given that long‐tailed tits live in kin neighbourhoods, this could simply be a consequence of the kin preference described above, but familiarity may also play a role. Sharp et al. (2005) and Napper and Hatchwell (2016) showed that social familiarity in the nest and in post‐fledging flocks influenced helping decisions, and Russell and Hatchwell (2001) found that failed breeders did not help at the nests of relatives that had spent the previous winter in a different flock to their own. Unfortunately, proximity cannot be readily distinguished from familiarity without detailed information on social association (Leedale, Li, & Hatchwell, 2020), e.g. through social network analysis, but these findings suggest that familiarity deserves further investigation as a driver of helping decisions.

Third, sex influences helping decisions, males being more likely to help than females (Leedale et al., 2018), as found in most avian cooperative breeding systems (Cockburn et al., 2017). The reason for this general pattern is not entirely resolved. In birds, adult sex ratios tend to be male‐biased (Donald, 2007) and dispersal tends to be female‐biased (Greenwood, 1980), increasing the likelihood that related non‐breeding males are available to help. In the case of long‐tailed tits, although there is an even adult sex ratio (Nam et al., 2011), females may have fewer helping opportunities because of their weaker kin structure (Figure 2) and looser kin associations than males (Napper & Hatchwell, 2016). Alternatively, or in addition to such effects, this sex biased helping could be influenced by body condition. The sexes contribute equally to nest‐building, but females produce a large clutch that weighs approximately 120% of their body mass and they incubate alone for c. 15 days with limited provisioning by males (Hatchwell et al., 1999). Therefore, by the time helping opportunities arise, female reproductive investment greatly exceeds that of males. Additionally, not all breeding males that failed and had an opportunity to help kin became helpers, and Meade and Hatchwell (2010) showed that males that spurned helping opportunities were late breeders with low survival prospects; they argued that this was because of their relatively poor body condition. Similarly, in an analysis of age‐related patterns of fitness in long‐tailed tits, Roper et al. (2022) reported that becoming a helper was positively affected by body condition in both sexes. However, although these studies are suggestive, we currently have insufficient information to test directly whether body condition per se drives sex‐differences in cooperation.

In summary, our principal conclusion from this section is that there is a range of population‐level (predation rate, length of season) and individual‐level (relatedness, familiarity, sex and body condition) factors determining the occurrence of cooperation. Further insights into helping decisions within kin neighbourhoods will need detailed, but hard to obtain information about prior social associations and the accuracy of information available to birds on their relatedness to others.

## 5 INCLUSIVE FITNESS CONSEQUENCES OF DISPERSAL DECISIONS

Ultimately, to understand the dispersal decisions animals make, we must quantify the fitness consequences for individuals that vary in their dispersal behaviour, and hence determine the selection pressures they experience. In this section, we review studies describing how variation in dispersal within and between sexes affects accrual of the direct and indirect components of inclusive fitness in long‐tailed tits. Most significantly, we show that the diverging fitness consequences of dispersal for males and females drive sexually antagonistic selection on this trait.

First, Green and Hatchwell (2018) used long‐term data (1994–2016) on lifetime reproductive success to compare the direct and indirect components of inclusive fitness of immigrant and philopatric (hereafter ‘resident’) males (*n* = 393) and females (*n* = 385) that recruited as breeders and whose complete life histories were known. A minority (37% of males, 36% of females) produced any fledglings in their lifetime, and an even smaller minority produced any recruits into the study population (19% of males, 21% of females). Thus, as reported by MacColl and Hatchwell (2004) and discussed above, fecundity was strongly skewed relative to the number of breeders in the population. Direct fitness was estimated from an individual's production of recruits in their lifetime after stripping away the effect of social partners, that is helpers (Hamilton, 1964), and accounting for extra‐pair paternity. The indirect fitness of helpers was estimated from the average effect of an individual helper on the productivity of breeders, multiplied by their genetic relatedness to the brood they helped (Green & Hatchwell, 2018).

**FIGURE 5:**
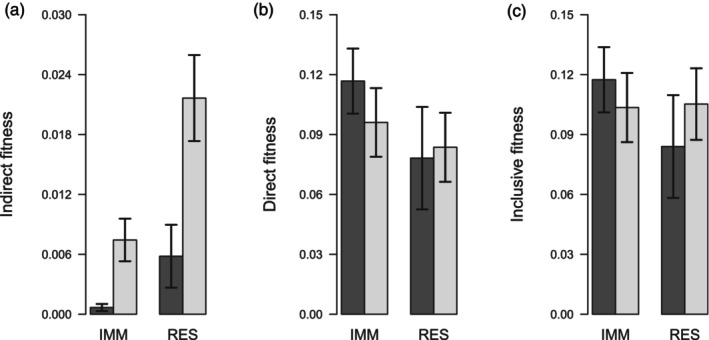
Mean ± SE (a) indirect, (b) direct and (c) inclusive fitness accrued by female (dark bars) and male (pale bars) long‐tailed tits given their immigration status (immigrants, IMM: 308 females, 211 males; residents, RES: 77 females, 182 males). From Green and Hatchwell (2018).

The accrual of fitness differed between residents and immigrants and between sexes (Figure 5). The indirect fitness of males and residents was significantly higher than that of females and immigrants (Figure 5a), reflecting the higher prevalence of helping among males than among females (Leedale et al., 2018) and the negative effect of dispersal on relatedness (Figure 2). In contrast, neither direct nor inclusive fitness differed in relation to dispersal status or sex (Figure 5b,c). Note that the differences in accrual of indirect fitness had little effect on inclusive fitness in this comparison because the contribution of indirect fitness to inclusive fitness is relatively small (13.4% for males, 1.5% for females).

**FIGURE 6:**
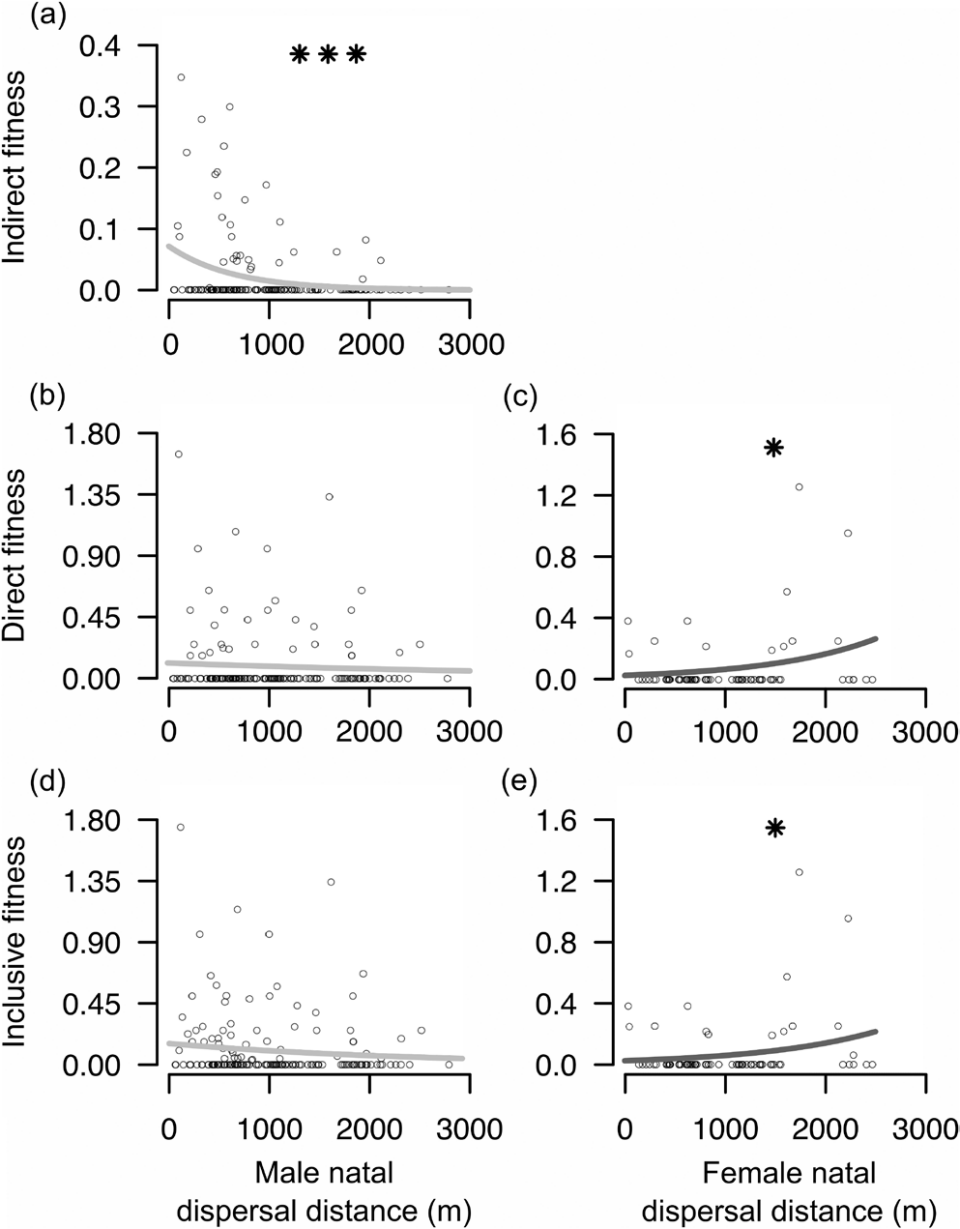
Relationship between fitness estimates and natal dispersal distances for 160 males (light grey; a, b, d) and 75 females (dark grey; c, e) that recruited within Rivelin Valley. Lines indicate (a) indirect, (b, c) direct and (d, e) inclusive fitness gained, and correspond to predictions from GLMMs of fitness given dispersal distance averaged across the cohort. Too few females accrued indirect fitness to derive regression lines for this component, but they were included in the inclusive fitness estimation. Dots show raw data. Stars indicate statistical significance: *p < 0.05, ***p < 0.001. Adapted from Green and Hatchwell (2018).

Second, Green and Hatchwell (2018) investigated the effect of natal dispersal distance within the 3km^2^ study site on the fitness of philopatric recruits, finding similar patterns as for the resident vs. immigrant comparison. Females gained very little indirect fitness regardless of dispersal distance, while male indirect fitness tended to decrease the farther they dispersed from their natal area (Figure 6a; Figure S1), again primarily due to a decline in the relatedness of helpers to recipients for more dispersive males. In contrast, the direct fitness of females increased the further they dispersed from their natal site (Figure 6c; Figure S1). The reason for this is uncertain but could be due to benefits of inbreeding avoidance (Leedale, Lachlan, et al., 2020; Leedale, Li, & Hatchwell, 2020; Leedale, Simeoni, et al., 2020). Overall, the inclusive fitness of males tended to decrease with dispersal distance while that of females tended to increase (Figure 6d,e; Figure S1). Thus, there was evidence for sexually antagonistic selection on dispersal in males and females, which was at least partly attributed to the fitness benefits of helping for philopatric males.

## 6 CONCLUSIONS

What does this review of studies into the ecological and demographic drivers of kin‐selected cooperation in long‐tailed tits tell us more broadly? We focus on four main conclusions relating to how population genetic structure arises, how social structure may be equally important in helping decisions, the role of predation as a driver of cooperation, and the value of social species for investigation of selection on dispersal strategies.

First, we have shown that three processes contribute to the emergence of kin neighbourhoods: limited dispersal, coordinated dispersal and a small effective population size. Population viscosity has long been viewed as a key driver of the transition to sociality, but when compared to non‐cooperative species the limited dispersal distances of long‐tailed tits are not atypical (Paradis et al., 1998; Russell, 1999). Dispersal of sibling coalitions has been reported in several other cooperatively breeding species (e.g., monk parakeets *Myiopsitta monachus*, Dawson Pell et al., 2021; acorn woodpeckers, Koenig et al., 2000; ground tits *Parus humilis*, Wang & Lu, 2014), but whether this is a more widespread phenomenon is an open question. Moreover, the fact that long‐tailed tits can maintain kinship ties across months of migratory movements and thus retain the capacity for kin‐directed cooperation is remarkable and contradicts the general expectation that migration precludes cooperative breeding (Arnold & Owens, 1998). Thus, stable groups of relatives living on permanent territories are not required for kin‐selected cooperation to evolve; indeed, mean relatedness in the kin neighbourhoods of long‐tailed tits is low (Figure 2) and substantially lower than in the social groups of most cooperative breeders (Green et al., 2016; Hatchwell, 2009). We suggest that a focus on population viscosity may neglect other demographic contributors to population genetic structure; fecundity bias may be just as important as dispersal behaviour in driving the emergence of kin neighbourhoods in long‐tailed tits, although we have yet to explore the relative contribution of each. More generally, while there has been extensive theoretical investigation of how multiple demographic processes contribute to the emergence of cooperation (e.g. Lehmann et al., 2006; Lehmann & Rousset, 2010), more empirical investigation of the demographic routes through which diverse forms of social organisation emerge in cooperatively breeding species is needed (e.g. Dyble & Clutton‐Brock, 2020).

Second, one of the conclusions of our review of the factors influencing the prevalence of helping is that a simple metric of relatedness measured at the population‐level did not predict cooperation across years or populations. This appears counterintuitive in a kin‐selected cooperative breeding system, but we think that it reflects the fact that individuals must make decisions about whether to help or not based on information they have about their own social environment. If kinship were the only important factor in such decisions, a direct link between kinship measure and cooperation would be expected. Yet, clearly, other factors matter at both individual and population levels. For example, it is easy to assume that animals have the necessary information to make adaptive decisions involving kin discrimination. However, our studies suggest that long‐tailed tits frequently make maladaptive choices in helping (Hatchwell et al., 2014) and mate choice (Leedale, Simeoni, et al., 2020), suggesting kin recognition errors. We are currently investigating whether such mistakes are caused by inaccurate information about relatedness to other members of the population. More specifically, we are examining what information about kinship is encoded in acoustic and olfactory cues, and also to what extent social associations at different life‐stages, assessed using social network analysis, influence behavioural decisions. This study system also demonstrates that close helper‐recipient relatedness is not required for kin selection to operate. Even though, on average, helpers have low relatedness to recipients (Green & Hatchwell, 2018; Nam et al., 2010) and indirect fitness represents a small component of inclusive fitness in long‐tailed tits (Figure 6; Hatchwell et al., 2014), this might be the only source of fitness an individual accrues in their lifetime (MacColl & Hatchwell, 2004). Thus, a kin‐selected route to cooperative breeding should not be dismissed simply because of low mean relatedness between social partners, or because a proportion of helpers assist non‐kin (Clutton‐Brock, 2002).

Third, this review has also revealed the key role that predation can play as a driver of cooperative breeding. Nest predators obviously have a pivotal role in causing fecundity bias, but they also generate failed breeders who can then redirect their care as helpers, the latter effect also being a function of the constrained breeding season that limits renesting opportunities. There is good evidence that predation is a constraint driving cooperative breeding, especially through cooperative defence (Bliard et al., 2024; Groenewoud et al., 2016), but predation risk is typically viewed as a constraint on dispersal rather than on the success of independent breeding attempts.

Finally, measurement of the fitness consequences of dispersal is a formidable challenge in any system, but the viscous populations of cooperatively breeding species provide rich opportunities to do so. The widely observed pattern of sex‐biased dispersal (Greenwood, 1980) is often assumed to arise from sexually antagonistic selection, but this has rarely been demonstrated quantitatively. Moreover, Green and Hatchwell (2018) showed that a key factor associated with this sexual disparity in dispersal was the indirect component of inclusive fitness that males accrued from kin‐directed cooperation. However, this analysis still leaves open questions. For example, while male philopatry was associated with greater indirect fitness benefits, some males dispersed away from their natal area (Figures 3, 5 and 6). This variation in male dispersal strategy could be determined by the availability of male kin with whom to disperse, but more detailed information is needed on how the size and association of sibling groups translates to dispersal movements. Similarly, it is surprising that some females remained close to their natal area (Figure 3), despite the apparent cost to their direct fitness (Figure 6). On the other hand, immigrant females did not have significantly higher fitness than residents (Figure 5), so selection for long‐distance dispersal may be weak. Again, it is likely that dispersal decisions were made according to females' social context, so more detailed monitoring of the timing and social consequences of association and dispersal are needed to provide further insights. The advent of new and increasingly miniaturised technologies for automated logging of marked individuals at fixed points (Beck et al., 2020; Capillo‐Lasheras et al., 2024) and for tracking through space and time (He et al., 2023) will facilitate such studies.

In conclusion, we suggest that a key factor underlying the advances described in this review is the long‐term, consistent nature of the study. There is strong advocacy for long‐term studies of natural populations (e.g. Clutton‐Brock & Sheldon, 2010), but they are often challenging to maintain. We also highlight the fact that although investigation of dispersal and quantification of fitness is facilitated in isolated populations, our findings suggest that study of open populations does not preclude these approaches, especially if individuals are relatively short‐lived. Lastly, while social complexity is often very appealing in the choice of species for studies in social evolution, we suggest that relatively simple systems, such as that of the long‐tailed tit, hold many advantages for disentangling the evolutionary, behavioural and ecological pathways to sociality.

## AUTHOR CONTRIBUTIONS

Ben J. Hatchwell conceived the ideas and lead the long‐term research project. Beth K. Woodward, Andrew F. Russell, Stuart P. Sharp, Ben J. Hatchwell collected the data; Beth K. Woodward, Jennifer Morinay analysed the novel data presented here; Jennifer Morinay and Ben J. Hatchwell led the writing of the manuscript. All authors contributed critically to the drafts and gave final approval for publication.

## CONFLICT OF INTEREST STATEMENT

The authors declare no conflict of interests.

## Supporting information


**Figure S1:** Relationship between fitness estimates and natal dispersal distances for 160 male (light grey; a, b, d) and 75 female (dark grey; c, e) long‐tailed tits that recruited within Rivelin Valley site. Lines indicate the probability to gain (a) indirect, (b, c) direct and (d, e) inclusive fitness, and correspond to predictions from GLMMs of fitness given dispersal distance averaged across the cohort. Boxplots are dispersal distances (central line: median value; outer box limits: first and third quartiles; horizontal dashed lines: approximately 2 SD around the interquartile range; circles: outliers). Stars indicate statistical significance: **p < 0.01; the point indicates marginal statistical significance: ⦁*p* < 0.1. Adapted from and see details in Green and Hatchwell (2018).

## Data Availability

Data available from the Dryad Digital Repository https://doi.org/10.5061/dryad.hhmgqnks3 (Morinay et al., 2024).
