# Supplementary material for: Ecological and demographic drivers of kin‐directed cooperation in a social bird: Insights from a long‐term study
Source: J Anim Ecol. 2025 Jan 28;94(4):485–500. doi: 10.1111/1365-2656.14237 (PMC11962233; doi:10.1111/1365-2656.14237)
Supplement: Supplementary file 1 — Figure S1: Relationship between fitness estimates and natal dispersal distances for 160 male (light grey; a, b, d) and 75 female (dark grey; c, e) long‐tailed tits that recruited within Rivelin Valley site. Lines indicate the probability to gain (a) indirect, (b, c) direct and (d, e) inclusive fitness, and correspond to predictions from GLMMs of fitness given dispersal distance averaged across the cohort. Boxplots are dispersal distances (central line: median value; outer box limits: first and third quartiles; horizontal dashed lines: approximately 2 SD around the interquartile range; circles: outliers). Stars indicate statistical significance: **p < 0.01; the point indicates marginal statistical significance: ⦁p < 0.1. Adapted from and see details in Green and Hatchwell (2018). [file JANE-94-485-s001.pdf]

Supporting Information

Ecological and demographic drivers of kin-directed cooperation in a social bird: insights from a long-term study.

- Journal of Animal Ecology (2025) –

Article doi https://doi.org/10.1111/1365-2656.14237

Jennifer Morinay[1*], Beth K Woodward[1,2], Andrew F Russell[3], Stuart P Sharp[4] & Ben J Hatchwell[1*]

[1]Ecology & Evolutionary Biology, School of Biosciences, University of Sheffield, Sheffield S10 2TN, UK

[2]Science and Technology Facilities Council, UKRI, Polaris House, Swindon SN2 1FL, UK

[3]Centre for Ecology and Conservation, University of Exeter, Penryn Campus, Cornwall TR10 9FE, UK

[4]Lancaster Environment Centre, Lancaster University, Lancaster LA1 4YQ, UK

***Correspondence**

Jennifer Morinay: j.morinay@sheffield.ac.uk

Ben J. Hatchwell: b.hatchwell@sheffield.ac.uk

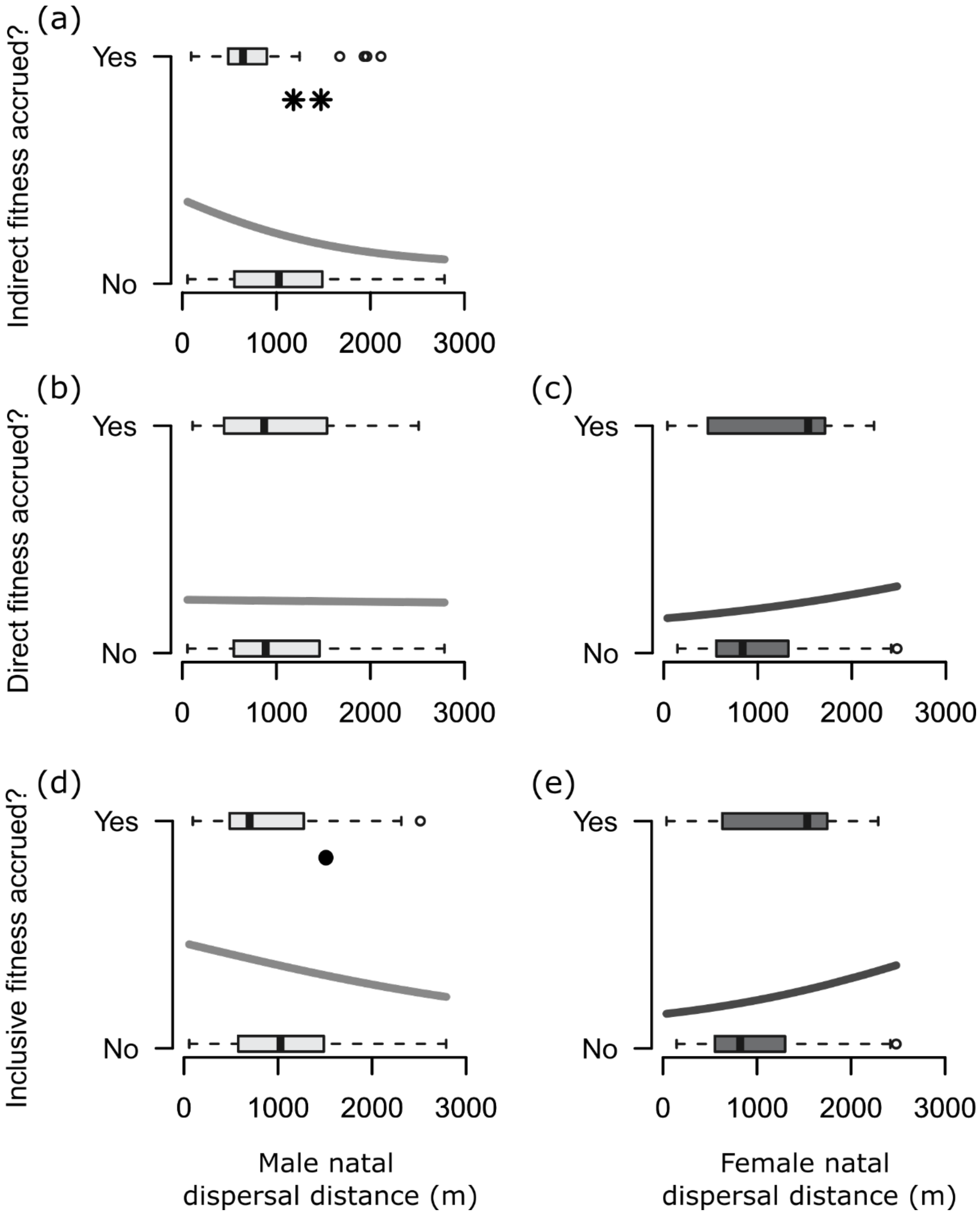

Figure S1. Relationship between fitness estimates and natal dispersal distances for 160 male (light grey; a,b,d) and 75 female (dark grey; c,e) long-tailed tits that recruited within Rivelin Valley site. Lines indicate the probability to gain (a) indirect, (b,c) direct and (d,e) inclusive fitness, and correspond to predictions from GLMMs of fitness given dispersal distance averaged across the cohort. Boxplots are dispersal distances (central line: median value; outer box limits: first and third quartiles; horizontal dashed lines: approximately 2 SD around the interquartile range; circles: outliers). Stars indicate statistical significance: **p<.01; the point indicates marginal statistical significance: • p<.1. Adapted from and see details in Green & Hatchwell (2018).

References

Green, J.P., & Hatchwell, B.J. (2018). Inclusive fitness consequences of dispersal decisions in a cooperatively breeding bird, the long-tailed tit (*Aegithalos caudatus*). *Proceedings of the National Academy of Sciences*, *115*(47), 12011–12016. https://doi.org/10.1073/pnas.1815873115
